# Analysis of the Correlation between MRI Imaging Signs and Lymphovascular Space Invasion in Endometrial Cancer

**DOI:** 10.2174/0115734056348172250407115649

**Published:** 2025-04-29

**Authors:** Chenwen Sun, Jiaying Mao, Yang Xia, Meiping Li, Zhenhua Zhao

**Affiliations:** 1 Department of Radiology, Shaoxing People’s Hospital (Shaoxing Hospital, Zhejiang University School of Medicine), Zhejiang University School of Medicine, Hangzhou 310058, China; 2 Department of Radiology, Shaoxing People’s Hospital (Shaoxing Hospital, Zhejiang University School of Medicine), Shaoxing, 312000, China; 3 Department of Radiology, Shaoxing Maternity and Child Health Care Hospital, Shaoxing, 312000, China; 4 Department of Pathology, Shaoxing Maternity and Child Health Care Hospital, Shaoxing, 312000, China

**Keywords:** Endometrial cancer, Lymphovascular space invasion, LVSI, MRI, MR diffusion-weighted imaging, Apparent diffusion coefficient

## Abstract

**Background:**

Determination of LVSI is the recommended criterion for performing lymphatic drainage and is important for the preoperative clinical decision-making process; however, Intraoperative Frozen Section (IFS) has limitations for the analysis of LVSI, and there is an urgent need for other indirect methods to predict the presence of LVSI.

**Aim:**

This study aimed to investigate the value of Magnetic Resonance Imaging (MRI) features in predicting Lymphovascular Space Invasion (LVSI) in endometrial cancer (EC).

**Objective:**

The objective of this study was to analyze MRI features that may be associated with LVSI and to explore their association.

**Methods:**

In this study, 179 patients who received treatment for EC confirmed by surgical pathology at two medical institutions from January 2017 to May 2024 were reviewed and grouped according to the presence or absence of vascular cancer embolism in the pathology. The MRI imaging features of the two groups were compared, including the maximum transverse diameter in the sagittal position, myometrial invasion, disruption of the uterine Junctional Zone (JZ), serosal surface, uterine appendages, cervical stromal invasion, lymph node enlargement, and its T2 value, and Diffusion-Weighted Imaging (DWI). The risk factors of the LVSI-positive group were determined by performing logistic regression analysis to analyze the correlation between Apparent Diffusion Coefficient (ADC) values and LVSI in EC.

**Results:**

There were 34 cases in the LVSI-positive group and 145 cases in the negative group. The maximum transverse diameter in sagittal position, myometrial invasion, interruption of the uterine JZ, serous surface, uterine appendages, cervical stromal invasion, lymph node enlargement, and their DWI and ADC values were statistically significant between the two groups (P < 0.05). In multivariate logistic regression analysis, lymph node enlargement (P = 0.001) and ADC value (P = 0.041) were identified as independent risk factors for positive LVSI.

**Conclusion:**

Lymph node enlargement and reduced ADC values (<0.767*10-3mm^2^/s) in MR imaging are of high value in predicting the occurrence of LVSI in patients with EC and can be used as an important reference for preoperative clinical diagnostic and therapeutic decisions for patients.

## 1 INTRODUCTION

Endometrial cancer (EC) is one of the most common malignant tumors affecting the female reproductive system in developed countries [1]. With the changing lifestyles of social groups, the prevalence of the disease has increased, posing a major threat to women's lives and health.

Surgery is typically the primary treatment for EC, with options including total hysterectomy and bilateral salpingo-oophorectomy, as well as lymph node assessment [2]. All EC patients should have a thorough lymphadenectomy, which includes the pelvic and para-aortic lymph nodes. However, multiple studies show that extensive lymphadenectomy has no effect on progression-free survival in low-risk EC patients. To avoid overtreatment, lymph node dissection should be performed more selectively and individually.

Lymphovascular space invasion (LVSI), also known as tumor lymphovascular infiltration, refers to the presence of tumor cells within channels bounded by endothelial cells. It is regarded as a critical phase in the metastatic progression of EC and is thought to be necessary for lymphatic dispersion [3]. Numerous investigations have found a strong link between LVSI, lymph node metastasis, and lymph node recurrence [4-6]. In 2015, the European Society of Gynaecological Oncology (ESGO) added the positive status of LVSI as a criterion for lymphadenectomy referral, even in the absence of any established histological risk factors [7]. LVSI, as an adverse prognostic factor in EC, is crucially important in determining its presence during preoperative clinical decision-making processes [3].

However, the limits of Intraoperative Frozen Section (IFS) analysis for LVSI make it difficult to definitively assess the status of LVSI before receiving the final pathology report. Pathological diagnosis of LVSI is also linked to the observer's experience [6, 8, 9]. Therefore, it is essential to establish indirect methods preoperatively or intraoperatively to predict the presence of LVSI. MRI in pelvic female systems detection offers non-invasive, clear, and accurate characteristics with high soft tissue resolution. The application of DWI can further provide additional diagnostic information [10]. Thus, MR imaging features may be helpful in predicting preoperative LVSI status to guide the choice of treatment strategy in patients with EC.

Therefore, the aim of this study is to analyze the MR imaging features that may be associated with LVSI and to investigate their correlation in order to provide a basis for the preoperative development of a rational treatment plan for patients with EC.

## 2 MATERIALS AND METHODS

### 2.1 Study Population

We retrospectively collected data from 179 female patients who underwent treatment for endometrial cancer (EC), confirmed by surgical pathology, between January 2017 and May 2024 at two medical institutions in Shaoxing City. This included 46 patients from Shaoxing People's Hospital (Centre A) and 133 patients from Shaoxing Maternity and Child Healthcare Hospital (Centre B), with a mean age of 59.7 ± 10 years. The ethics committees of all participating institutions approved this retrospective study, and all waived the requirement for written informed consent from patients. The study was conducted in accordance with the principles of the Declaration of Helsinki.

Inclusion criteria included (1) patients with EC confirmed by postoperative pathology and (2) patients with complete clinical data and, all of whom underwent preoperative MRI examination. Exclusion criteria included: (1) an interval of more than 2 weeks between the preoperative MRI examination and surgery; (2) cases where the attending physician assessed the image quality as poor due to artifacts, preventing clear visualization of the lesion's edges or resulting in incomplete MRI sequences; and (3) patients with other concurrent tumors or a history of tumor radiotherapy, chemotherapy, or hormone therapy.

### 2.2 MRI Acquisition

MRI was acquired using a 3.0 T magnetic resonance scanner (Siemens Verio, Germany) in center A and a 1.5 T magnetic resonance scanner (Siemens Avanto, Germany) in center B. All patients were required to breathe freely in the supine position during data acquisition. The following sequences were acquired: T1WI, T2WI transverse axial, coronal, and sagittal pressure-lipid sequences, contrast-enhanced T1-weighted imaging (CE-T1WI), and axial DWI, accompanied by an ADC map, with a b-value of 0 and 1000 s/mm^2^. The primary scanning sequences in center A included T2WI (TR/TE: 3.48 ms /1.3 ms, FOV: 320 × 320 mm), CE-T1WI (TR/TE: 6700 ms, FOV: 288 × 288 mm), DWI (TR/TE: 6700 ms/92 ms, FOV: 160 × 160 mm), and ADC (TR/TE: 6700 ms/92 ms, FOV: 160 × 160 mm). The main scanning sequences in the B center included T2WI (TR/TE: 5210 ms/79 ms, FOV: 320 × 320 mm), CE-T1WI (TR/TE: 735 ms/11 ms, FOV: 320 × 320 mm), DWI (8900 ms/84 ms, 160 mm × 136 mm), and ADC (TR/TE: 8900 ms/84 ms, FOV: 160 mm × 136 mm).

### 2.3 MRI Observation and Diagnostic Criteria

A double-blind method was used, in which 2 associate physicians with 7 years of experience in pelvic MRI diagnosis read the films to observe the characteristics of the overall uterine impact and signal characteristics and the MRI manifestations of EC with different invasive ranges. MRI images and corresponding pathological images of the typical case are shown in Fig. (**1**).

All measurements were viewed and taken directly at the workstation provided with the Siemens magnetic resonance imaging equipment, using the tools included with the system. Sketches and calculations were first made by a radiologist (Reader 1) with 7 years of experience in gynecological imaging. One week later, Reader 2, who also had 7 years of experience in diagnostic pelvic MRI, randomly selected 30 patients to be sketched and calculated again. Neither radiologist obtained clinical or pathological information about any of the patients. Intra- and inter-class correlation coefficients (ICC) were used to assess inter-observer agreement and reproducibility.

#### 2.3.1 Overall Imaging Features and Signal Characteristics of EC

The overall image features of the EC lesion were observed to identify the best measurement level. Three regions of interest (ROI), each with a diameter of 3 mm, were selected within the T2-weighted, DWI (b=1000 s/mm^2^), and ADC images. These regions were measured and averaged, ensuring that the peri-endometrial binding zone and the fluid in the uterine cavity were avoided. The best measurement level in the sagittal plane was identified,and the maximum transverse diameter of the sagittal lesion and the maximum transverse diameter of the uterus were measured, and they were divided to get the percentage of myometrial invasion (%).

#### 2.3.2 Other Diagnostic Criteria for EC

When observing T2WI images, the focus was on evaluating the integrity of the endometrial junctional zone (JZ) and serous surface, as well as the early dynamic enhancement of the JZ. EC lesions can extend from the uterine corpus to the cervix, characterized by discontinuous or interrupted low-signal stroma band in the cervix and cervical canal dilation, indicating cervical invasion. Irregular morphology of affected uterine adnexa revealed abnormal nodules or masses on enhancement scans. Further, affected lymph nodes exhibited isointense signals on T1WI and T2WI, with mild enhancement on routine scans, typically considering lymph node enlargement (>10mm diameter) as indicative of lymphadenopathy. Signal changes were also observable on DWI.

### 2.4 Statistics

We conducted a statistical analysis of the results using SPSS 27.0 software, presenting metric data as mean ± standard deviation. Fisher's exact test and chi-square test were used for categorical variables. For continuous variables, a t-test or Mann-Whitney test was applied, with P < 0.05 indicating statistical significance. Subsequently, univariate logistic regression analysis identified imaging features associated with LVSI-positive status. Multivariable logistic regression then estimated Odds Ratios (ORs) for each covariate. Variables with P < 0.05 in univariate analysis were included in multivariate analysis. Receiver Operating Characteristic (ROC) curve analysis assessed the accuracy of identified risk factors in predicting LVSI in EC patients, with Youden's index used to determine optimal cutoff values. Using these risk factors, we developed a predictive model for LVSI and further evaluated its performance through ROC curve analysis. The processes of model evaluation were conducted using the R software (version 4.3.1).

## 3 RESULTS

### 3.1 Baseline Characteristics of the Patients

**Fig. (2):**
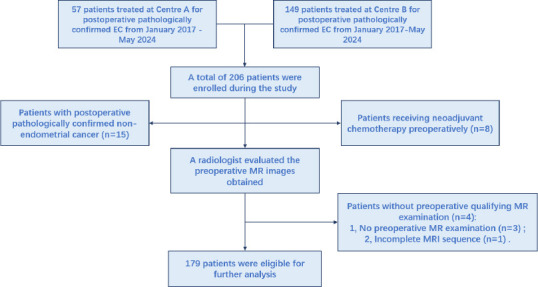
Workflow shows the selection of the studying population and exclusion criteria.

A total of 206 patients underwent surgical treatment for EC during the study period. Patients with non-endometrial cancer (n=15), patients who received neoadjuvant chemotherapy (n=8), and patients who did not have preoperative qualifying MR examinations (n=4) were excluded. A total of 179 patients were included in the final analysis (Fig. **2**). Of these, 34 (19%) were positive for LVSI and 145 (81%) were negative. The distribution of variables in the two groups is shown in Table **1**. The ICC for each group of observations was greater than 0.75, indicating high inter-observer agreement. LVSI-positive patients had larger lesions (24.6 *vs*. 17.3 mm, P<0.001), and deeper infiltration of lesions (51.9 *vs*. 38.4%, P<0.001), interrupted uterine JZ (P=0.047), serous surface invasion (P=0.004), uterine appendages invasion (P=0.002), cervical stromal invasion (P = 0.018), and peripheral lymph node enlargement (P < 0.001) were more common, with higher DWI signals (66.8 *vs*. 57.2, P < 0.001) and lower ADC values (0.733 *vs*. 0.830*10-3 mm^2^/s, P < 0.001) compared with LVSI-negative patients. There was no statistical difference in T2 values between the two groups (P > 0.05). Moreover, specific details of the pathology of the 179 patients included during the study period are detailed in the Appendix.

### 3.2 Univariate and Multivariate Logistic Analysis

The univariate and multivariate logistic analyses of MRI imaging features are shown in Table **2**. In unifactorial analysis, the maximum transverse diameter in sagittal position (P < 0.001), myometrial invasion (P < 0.001), serous surface (P = 0.025), uterine appendages (P = 0.011), cervical stromal invasion (P = 0.022), lymph node enlargement (P < 0.001), high DWI signal (P < 0.001), and low ADC value (P < 0.001) were identified as risk factors for LVSI. In multifactorial logistic regression analysis, enlarged lymph nodes (P = 0.001) and low ADC value (P = 0.041) were identified as risk factors for LVSI. The remaining factors were not significant.

### 3.3 Risk factor ADC values and LVSI

ADC value (P=0.041) was identified as a risk factor for LVSI. The ROC curve for the prediction of LVSI by ADC value is shown in Fig. (**3**). The model had a sensitivity of 71.0% and a specificity of 67.7% at the optimal cut-off value of 0.767*10-3 mm^2^/s. In the ROC curve analysis, the area under the curve (AUC) was 0.727. Decreased ADC values (<0.767 × 10^-3 mm^2^/s) held higher predictive value for LVSI occurrence in patients with EC.

### 3.4 Development and Evaluation of a Prediction Model for LVSI

**Fig. (4):**
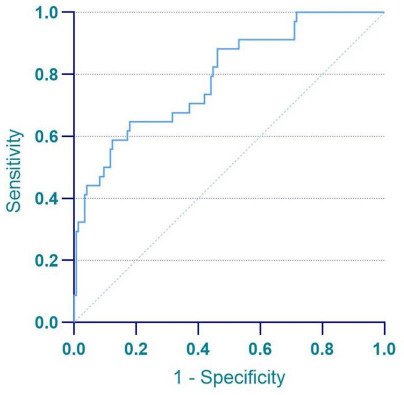
The ROC curves of the model in predicting LVSI on the training cohort.

Based on the results of univariate logistic regression analysis, we have developed a simplified LVSI prediction model for clinical use. This model incorporates eight risk factors associated with LVSI, including the maximum trans-verse diameter in sagittal position, myometrial invasion, serous surface invasion, uterine appendages invasion, cervical stromal invasion, lymph node enlargement, DWI value, and ADC value. We calculated the LVSI for all patients in the study group and used ROC curve analysis to evaluate the diagnostic accuracy (Fig. **4**), yielding an AUC of 0.790 (95% CI=0.704-0.876; sensitivity 64.7%; specificity 82.1%).

**Fig. (5):**
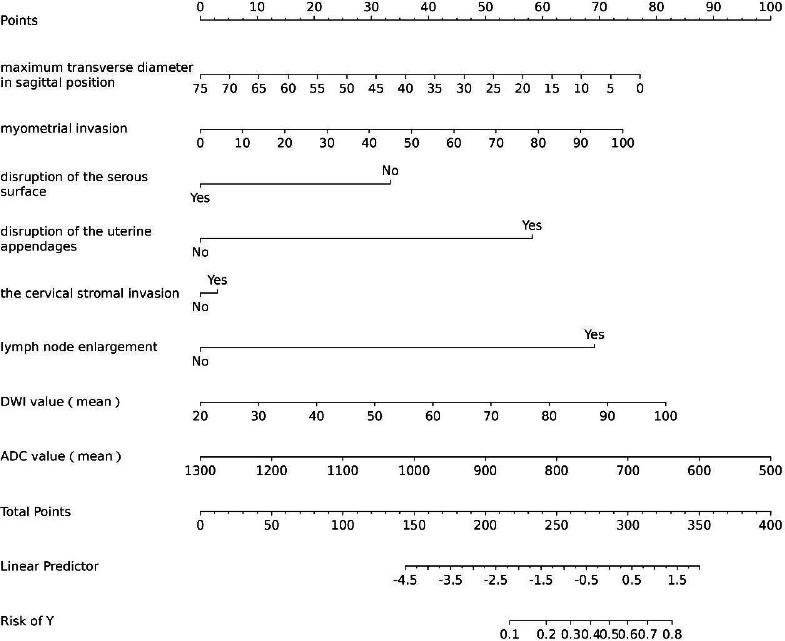
The nomogram plots, based on the training cohort data, illustrate the nomogram scores of the LVSI prediction model, displaying predicted recurrence risk probabilities.

To better assess the clinical application value of the model, we further validated the constructed LVSI prediction model. We created a nomogram and performed calibration curve analysis and Decision Curve Analysis (DCA). The nomogram (Fig. **5**) can transform a complex model into an intuitive and understandable graphic, making the results of the prediction model more readable.

**Fig. (6):**
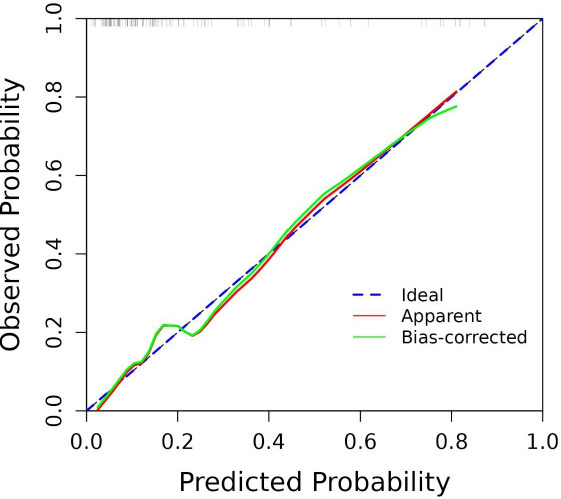
Calibration curve analysis of LVSI prediction models on the training cohort.

**Fig. (7):**
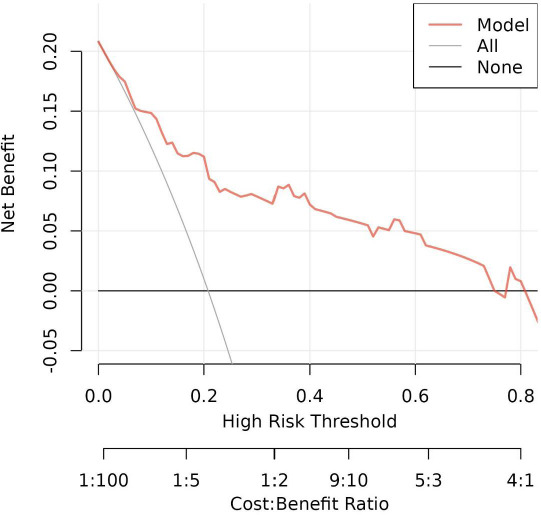
Decision curve analysis of the LVSI prediction model on the training cohort.

The numerical values of each risk factor are projected onto the scale to obtain the score for each item, and these scores are summed to determine the total score. The higher the total score, the greater the probability that the endometrial cancer patient has a positive LVSI. Moreover, the calibration curve analysis (Fig. **6**) and DCA (Fig. **7**) indicated that the model has good calibration and clinical applicability in predicting LVSI risk.

## 4 DISCUSSION

In our study, we assessed the correlation between the occurrence of LVSI in EC and its MRI features. Preliminary results indicated that the presence of LVSI is associated with the maximum transverse diameter in the sagittal position, myometrial invasion, serosal surface, uterine appendages, cervical stromal invasion, lymph node enlargement, DWI value, and ADC value of the lesion. Furthermore, multivariable logistic regression analysis revealed that lymph node enlargement and ADC values have significant potential for preoperative prediction of LVSI in EC patients. Our findings reveal that lesions exhibiting more restricted diffusion and aggressive characteristics on MRI are more likely to be associated with LVSI. Additionally, this study developed a risk assessment model incorporating LVSI-related risk factors to predict the likelihood of LVSI occurrence in EC patients. The results indicate that the model demonstrates high discriminative efficiency and clinical applicability in predicting the risk of LVSI.

Many previous studies [11-15] have confirmed that LVSI is an adverse prognostic factor in EC. LVSI is considered the strongest independent risk factor for reduced survival and distant recurrence, suggesting its potential role in disease progression through hematogenous dissemination. The LVSI status also affects the decision-making process for lymphadenectomy. Several studies [16, 17] have shown that low-risk EC patients do not benefit from lymphadenectomy, whereas for intermediate to high-risk EC patients, lymphadenectomy is associated with improved overall survival [18, 19]. Therefore, pelvic and para-aortic lymphadenectomy is recommended for intermediate to high-risk patients. Thus, it is essential to clearly stratify EC patients into low-risk and intermediate to high-risk categories. The presence or absence of LVSI plays a key role in distinguishing low-risk from intermediate to high-risk EC. According to the revised ESMO classification, the absence of LVSI categorizes it as low-risk, while the presence of LVSI categorizes it as intermediate-risk [20]. The 2023 International Federation of Gynecology and Obstetrics (FIGO) staging for EC also points out that non-invasive histological types of EC, regardless of myometrial invasion depth or cervical stromal involvement, are classified as FIGO stage II if there is extensive LVSI.

Although LVSI status is crucial for risk assessment and decision-making for the treatment, it is typically not determined until the final pathological report is available. Several studies [6, 21-23] have evaluated the accuracy of Intraoperative Frozen Section (IFS) in determining LVSI status. Kumar *et al.* noted that patients initially classified as low-risk EC through IFS often experience significant disease upstaging and upgrading upon final pathological examination [21]. Additionally, 31.7% of patients initially classified as LVSI-negative by IFS were found to be LVSI-positive in the final pathology examination. Turan *et al.* reported that IFS had only 50% sensitivity in identifying LVSI [22]. Factors contributing to inaccurate IFS analysis include interpretive variability due to experience, technical artifacts introduced by frozen section techniques, and inadequate sampling due to selective sampling [6, 24]. Therefore, comprehensive evaluation to predict LVSI status is crucial for the individualized treatment of EC patients.

MRI, like histopathology, provides crucial supportive data for determining the clinical extent and approach of surgery. Multi-modal MRIs are of significant value in preoperatively predicting the pathological grading and staging of EC. The signal intensity of EC on MRI can be used to predict tumor invasiveness [25-27]. When MRI shows low-risk features, such as moderate tumor size, limited myometrial invasion, no restricted diffusion, and no suspicious lymph nodes, it may guide less aggressive resection of early-stage tumors to reduce the incidence of complications. We found that lymphade-nopathy on MRI (P=0.001) was identified as a risk factor for LVSI, consistent with previous research showing a significant association between the presence of LVSI and lymph node invasion [4, 28]. It is hypothesized that the impact of LVSI on survival is due to its association with lymph node metastasis, as EC is thought to spread to regional lymphatic vessels and then to regional lymph nodes and, indeed, throughout the body.

The increased cell density in tumor tissue restricts the Brownian motion of water molecules, and this restriction can be measured using ADC values. ADC is negatively correlated with tumor cell density and is used to distinguish between benign and malignant lesions, determine tumor grading and size, and evaluate tumor response to treatment. Some studies suggest that ADC values can help determine the grading of EC [29-31]. The research by Keven *et al.* shows that measuring ADC values can be used to detect LVSI and potentially predict cancer behavior [32]. Nougaret *et al.* demonstrated a correlation between the presence of LVSI and lower histological ADC [29].

Zhang *et al.* also found significantly lower ADC values in tumor lesions with LVSI compared to those without LVSI [33], which is consistent with our research findings. In our study, we confirmed that low ADC values (P = 0.041) are a risk factor for LVSI and demonstrated that a decrease in ADC values is highly predictive of the occurrence of LVSI in EC patients.

In addition, based on the results of risk factor analysis, we developed a simple LVSI prediction model for clinical use. We collected data sets from two independent institutions to enhance the reliability and generalizability of the model's predictive performance. The prediction model demonstrated a good AUC value (AUC=0.790) in the training set, indicating good predictive performance. It also showed good calibration and clinical applicability in calibration curve and decision curve analysis, reflecting its generalization ability and robustness.

This study has several limitations that need to be addressed. Firstly, it is a retrospective study with a relatively small sample size, particularly among patients with EC confirmed pathologically as positive for LVSI. The measurement of ADC values was based on manually delineated ROI without texture analysis. Despite these biases, statistically significant results were still found. It is acknowledged that further validation is necessary for future investigations using independent datasets with larger patient cohorts. Another limitation is that obtaining endometrial sampling *via* hysteroscopy biopsy or curettage before acquiring MRI sequences may affect tumor volume, and bleeding or fluid accumulation could also influence tumor signal characteristics. Future studies should aim to track patient histories comprehensively on a large scale to mitigate these confounding factors.

## CONCLUSION

Our study found a significant correlation between observed lymphadenopathy on MR imaging and ADC values with vascular invasion in EC. These findings can assist in preoperatively identifying intermediate to high-risk patients with vascular invasion, providing objective evidence to guide comprehensive clinical management and assess patient prognosis.

Continuous variables were presented as mean ± standard deviation, while categorical variables were reported as patient numbers with percentages in parentheses. Numbers marked in bold indicate p-values less than 0.05, which is considered statistically significant.

Graphical abstract of the study. In our study, we included 179 EC patients with postoperative pathological diagnosis of LVSI, evaluated the correlation between the occurrence of LVSI in EC and its MRI features, and preliminarily screened for relevant high-risk factors. Based on multifactorial logistic regression analysis, lymph node enlargement, and ADC values were of potentially important significance, and a risk assessment model was also constructed. The results showed that the model demonstrated high discriminatory efficiency in predicting the occurrence of LVSI.

A 55-year-old female patient whose pathology showed well-differentiated endometrial carcinoma with LVSI.A: CE-T1WI image; B: T2WI image; C: Immunohistochemical staining image of the patient's postoperative pathology blocked section stained with mTOR protein. The magnification of the images is 10×40; D: DWI image; E: ADC image.

mTOR (mammalian target of rapamycin) is an atypical serine/threonine protein kinase belonging to the phosphatidylinositol kinase-related kinase (PIKK) family. It is an important signaling pathway protein closely related to cell growth and proliferation. Positive staining of mTOR usually appears in the cytoplasm and nucleus. The mutation rate of the mTOR pathway in endometrial cancer is high, and the cancerous cells show strong positivity in the stained sections. In Image C, strongly positive tumor cell clusters can be observed in the lumen lined by endothelial cells, indicating the presence of LVSI.

The blue line is the ideal calibration curve, i.e., the predicted value is equal to the actual value. The closer the model calibration curve is to this line, the better the predictive ability of the model.

The horizontal axis represents the risk of disease occurrence, the vertical axis represents the net income rate of the patients, and the grey slash represents that all patients have an adverse outcome. When the predictive model curve (red) is higher than the grey slash, it represents that the corresponding patient can benefit.

## Figures and Tables

**Fig. (1):**
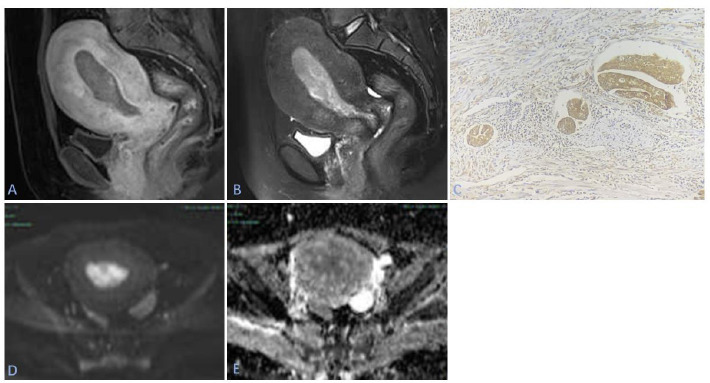
MRI images and corresponding pathological images of a typical case: (**A**) CE-T1WI image; (**B**) T2WI image; (**C**) Immunohistochemical staining image of the patient's postoperative pathological blocked section stained for mTOR protein. The magnification of the image is 400×; (**D**) DWI image; (**E**) ADC image.

**Fig. (3):**
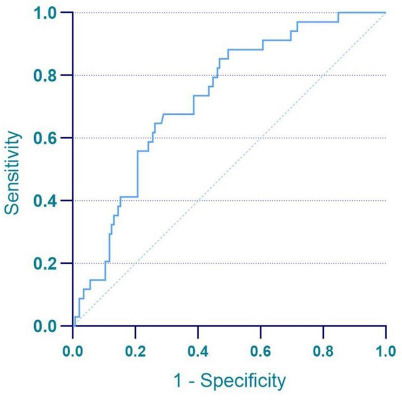
The ROC curves of ADC in predicting LVSI.

**Table 1:** LVSI status and MRI features in EC.

**Characteristics**	**LVSI+ (%)**	**LVSI- (%)**	**P-value**
**Total**	34(19.0)	145(81.0)	
**Maximum transverse diameter in sagittal position**	24.6mum t	17.3mum t	<0.001
**Myometrial invasion(%)**	51.9etria	38.4etria	<0.001
**JZ interruption**			
**-**	1(0.6)	23(12.8)	0.047
**+**	33(18.4)	122(68.2)	
**Serous surface invasion**			
**-**	31(17.3)	144(80.4)	0.004
**+**	3(1.7)	1(0.6)	
**Uterine appendages invasion**			
**-**	30(16.8)	143(79.9)	0.002
**+**	4(2.2)	2(1.1)	
**Cervical stromal invasion**			
**-**	26(14.5)	132(73.7)	0.018
**+**	8(4.5)	13(7.3)	
**lymph node enlargement**			
**-**	21 (11.7)	136(76.0)	<0.001
**+**	13(7.3)	9(5.0)	
**T2 value (mean)**	396.6±63.3	390.5±72.2	0.655
**DWI (mean)**	66.8±14.5	57.2±11.9	<0.001
**ADC (mean)**	0.733±0.102	0.830±0.126	<0.001

LVSI: lymphovascular space invasion; JZ, junctional zone, is the normal structure in the uterine under MR imaging.

**Table 2:** Univariate and multivariate logistic analysis of MRI features in EC.

**Characteristics**	**Univariate Analysis**								**Multivariate Analysis**						
	OR				95% CI		P		OR			95% CI		P	
**Maximum transverse diameter in sagittal position**	1.060				(1.025-1.098)		<0.001		0.978			(0.904-1.059)		0.584	
**Myometrial invasion(%)**	1.037				(1.016-1.058)		<0.001		1.018			(0.974-1.064)		0.433	
**JZ interruption**	-				-		-		-			-		-	
**-**	1(Ref)				-		-		-			-		-	
**+**	6.221				(0.810-47.784)		0.079		-			-		-	
**Serous surface invasion**	-				-		-		-			-		-	
**-**	1(Ref)				-		-		1(Ref)			-		-	
**+**	13.935				(1.402-138.468)		0.025		1.359			(0.065-28.262)		0.843	
**Uterine appendages invasion**	-				-		-		-			-		-	
**-**	1(Ref)				-		-		1(Ref)			-		-	
**+**	9.533				(1.669-54.446)		0.011		4.612			(0.542-39.225)		0.162	
**Cervical stromal invasion**	-				-		-		-			-		-	
**-**	1(Ref)				-		-		1(Ref)			-		-	
**+**	3.124				(1.177-8.291)		0.022		1.835			(0.527-6.391)		0.341	
**lymph node enlargement**	-				-		-		-			-		-	
**-**	1(Ref)				-		-		1(Ref)			-		-	
**+**	9.354				(3.560-24.582)		<0.001		7.630			(2.264-25.716)		0.001	
**T2 value (mean)**	1.001				(0.996-1.007)		0.653		-			-		-	
**DWI (mean)**	1.060				(1.028-1.094)		<0.001		1.028			(0.989-1.068)		0.161	
**ADC (mean)**	0.993				(0.990-0.997)		<0.001		0.995			(0.991-1.000)		0.041	

**Note:** Covariates with P<0.05 on univariate analysis were included in the multivariate model. OR, odds ratio; CI, confidence interval; Ref, reference.

## Data Availability

The datasets used during the current study are available from the corresponding author [Z.Z] upon request.

## LIST OF ABBREVIATIONS

IFS: Intraoperative frozen section
LVSI: Lymphovascular space invasion
EC: Endometrial cancer
JZ: Junctional zone
ADC: Apparent diffusion coefficient
EC: Endometrial cancer
ICC: Inter-class correlation coefficients

## STANDARDS OF REPORTING

STROBE guidelines were followed.

## APPENDIX

Information of the samples included
